# Preferential FGF18/FGFR activity in pseudoglandular versus canalicular stage human lung fibroblasts

**DOI:** 10.3389/fcell.2023.1220002

**Published:** 2023-08-28

**Authors:** Randa Belgacemi, Caroline Cherry, Imad El Alam, Andrew Frauenpreis, Ian Glass, Saverio Bellusci, Soula Danopoulos, Denise Al Alam

**Affiliations:** ^1^ Lundquist Institute for Biomedical Innovation at Harbor-UCLA Medical Center, Torrance, CA, United States; ^2^ Department of Pediatrics, University of Washington School of Medicine, Seattle, WA, United States; ^3^ Excellence Cluster Cardio-Pulmonary System (ECCPS), Universities of Giessen and Marburg Lung Center (UG-MLC), Justus-Liebig-University Giessen, German Center for Lung Research (DZL), Giessen, Germany; ^4^ Department of Pediatrics, David Geffen School of Medicine at UCLA, Los Angeles, CA, United States

**Keywords:** FGF18, FGFR, lung development, mesenchyme, progenitor cells

## Abstract

Fibroblast growth factor (FGF) signaling is necessary for proper lung branching morphogenesis, alveolarization, and vascular development. Dysregulation of FGF activity has been implicated in various lung diseases. Recently, we showed that FGF18 promotes human lung branching morphogenesis by regulating mesenchymal progenitor cells. However, the underlying mechanisms remain unclear. Thus, we aimed to determine the role of FGF18 and its receptors (FGFR) in regulating mesenchymal cell proliferation, migration, and differentiation from pseudoglandular to canalicular stage. We performed siRNA assays to identify the specific FGFR(s) associated with FGF18-induced biological processes. We found that FGF18 increased proliferation and migration in human fetal lung fibroblasts (HFLF) from both stages. FGFR2/FGFR4 played a significant role in pseudoglandular stage. HFLF proliferation, while FGFR3/FGFR4 were involved in canalicular stage. FGF18 enhanced HFLF migration through FGFR2 and FGFR4 in pseudoglandular and canalicular stage, respectively. Finally, we provide evidence that FGF18 treatment leads to reduced expression of myofibroblast markers (ACTA2 and COL1A1) and increased expression of lipofibroblast markers (ADRP and PPARγ) in both stages HFLF. However, the specific FGF18/FGFR complex involved in this process varies depending on the stage. Our findings suggest that in context of human lung development, FGF18 tends to associate with distinct FGFRs to initiate specific biological processes on mesenchymal cells.

## Introduction

The human lung is a complex system that undergoes organogenesis through a series of tightly regulated interactions between various cell types and signaling pathways (Shi et al., 2007; Herriges and Morrisey, 2014; Hines and Sun, 2014; Caldeira et al., 2021). Among these pathways, the fibroblast growth factor pathway, comprising ligands (FGFs) and receptors (FGFR1, 2, 3 and 4), is known to play critical roles in lung development, such as regulating cell growth, proliferation, and differentiation (Volckaert and De Langhe, 2015; Jones et al., 2020b; Yang et al., 2021). Furthermore, aberrant FGF signaling is associated with various lung diseases, including idiopathic pulmonary fibrosis and lung cancer (Colvin et al., 2001; Hegab et al., 2019; Yang et al., 2021). Most studies on the FGF pathway have been conducted using mouse models. However, we have demonstrated significant discordances in the effects of FGF signaling between humans and mice (Danopoulos et al., 2019a; Danopoulos et al., 2019b). Our studies show that, unlike in mouse, FGF10 is unable to induce branching in human fetal lungs (Danopoulos et al., 2019b). Rather, we showed that FGF18, whose expression increases throughout lung development, promotes branching morphogenesis *ex vivo* in human lung explants and regulates mesenchymal progenitor cell commitment in the lung (Danopoulos et al., 2023). Although FGF18 has been shown to bind to at least one isoform from each of the FGFRs (FGFR1, FGFR2, FGFR3, and FGFR4), previous studies have suggested that its effects on lung are mainly mediated by FGFR2, FGFR3, and FGFR4 (Whitsett et al., 2002; Davidson et al., 2005; Brown et al., 2014; Jones et al., 2020a), all of which are expressed by fetal lung mesenchymal cells (Danopoulos et al., 2023).

During human lung development, mesenchymal cells are important regulators of branching morphogenesis, alveolarization, and differentiation of other cell types (Slavkin et al., 1984; Waszak and Thébaud, 2011; Danopoulos et al., 2020; Nasri et al., 2021). We and others have shown that FGF18 plays a critical role in determining mesenchymal cell fate (Jeon et al., 2012; Song et al., 2018; Danopoulos et al., 2023). However, our understanding of how FGF18 regulates mesenchymal cell processes during human lung development, and through which receptors this occurs, remains elusive. In this study, we sought to investigate the role of the FGF18/FGFR in fetal lung mesenchymal cells derived from pseudoglandular and canalicular stage.

## Materials and methods

### Study approval

The human fetal lung tissues used in this study were collected under IRB approval (The Lundquist Institute 18CR-32223-01) provided to the lab by the University of Washington Birth Defects Research Laboratory. They are de-identified and the only information collected were gestational age and known lung pathologies. Informed consent was provided for each lung collected and used in this study.

### Human fetal lung fibroblasts culture

Fresh human fetal lung tissues between 12 and 21 weeks gestation (Table 1) were mechanically and enzymatically dissociated by treatment with DNAse I (8U/mL, 79256, Quiagen, GE) and collagenase IV (2 mg/mL, 17104-019, Gibco, MA, USA) to obtain primary lung fibroblasts. Fibroblasts were cultured by differential adhesion in Dulbecco’s Modified Eagle’s Medium: nutrient mix F-12 (D-MEM/F-12) with 1% FBS. Culture media was changed every 48 h and cells were kept in a cell culture incubator at 37°C, 5% CO2. Cells were plated in 12 wells plates or 4 wells chamber slides (respectively at 6 × 10^4^ and 3.5 × 10^4^ cells per well) until 60% confluency, then starved during 24 h before any treatment or assay. For all experiments, each “n” indicates a biological replicate.

**TABLE 1:** Fetal human lung samples.

Gestational age (weeks)	Sex
12.4	F
12.1	F
12.4	M
12.1	M
20.6	M
20.6	F
19.6	M
20.1	F

### siRNA assay

siRNA pool for *FGFR2, FGFR3* and *FGFR4* (Dharmacon, CO, USA), detailed in Table 2, were incubated with lipofectamine (Lipofectamine RNAimax, Invitrogen, CA, USA) in D-MEM/F-12 with no serum or antibiotics and allowed to complex for 5 min at room temperature. The complex was added to the cell culture media at a final siRNA pool concentration of 10 nM for each and incubated for 24 or 48 h in the absence or presence of rhFGF18 (100 ng/mL, # 8988-F18, R&D, MN, USA).

**TABLE 2:** siRNA.

Gene	Cat number
FGFR2	L-003132-00-0010
FGFR3	L-003133-00-0010
FGFR4	L-003134-00-0010

### Real-time PCR analyses

RNA was extracted using the iNtRon Biotechnology, Inc. Easy-Spin™ Total RNA Extraction Kit (Burlington, MA, USA). RNA was reverse transcribed into cDNA using the Tetro cDNA Synthesis Kit (Bioline, Taunton, MA, USA) according to the manufacturer’s instructions. PCR products were amplified using specific TaqMan gene expression assays (listed in Table 3), Applied Biosystems, Foster City, CA, USA) and the TaqMan Universal PCR Master Mix II (Applied Biosystems). PCR products were detected using the StepONE Plus Real Time PCR System (Applied Biosystems). Each sample was run in triplicate.

**TABLE 3:** Taqman^TM^ probes used for RT-qPCR.

Target gene	Probe ID
*ACTA2*	HS00426835
*ETV4*	HS00383361
*ETV5*	HS00927578
*GAPDH*	HS02786624
*ADRP*	Hs00605340
*COL1A1*	Hs00164004
*PPARG*	Hs01115513
*MKI67*	Hs04260396
*FGF18*	Hs00826077

### Immunofluorescence (IF) staining

Cells were washed with PBS, fixed in 4% PFA at room temperature (RT) for 30 min and blocked in 3% bovine serum albumine/5% Normal Goat Serum/0.1% Triton-X100 in TBS for 2 h. Primary antibodies (detailed in Table 4) were added overnight at 4°C. The next day slides were washed in PBS, stained with species appropriate secondary antibodies for 1 h at room temperature, and counterstained with DAPI.

**TABLE 4:** Antibodies list.

Antibodies	RRID	Application
ACTA2	AB_2223500	WB (1/500)
		IF (1/200)
KI67	AB_2341197	IF (1/200)
ADRP	AB_2919116	WB (1/500)
GAPDH	AB_561053	WB (1/1000)
P-ERK	AB_2315112	WB (1/1000)

### Quantitative analysis of proliferation

Cells from chamber slides were immunostained for KI67 (Table 4) and imaged using a ×40 objective. Ten images were captured and quantified per sample. Cells were quantified using HALO® Image Analysis Platform (version 3.4.2986, Highplex FL and Area quantification module, Indica Labs, Inc; Albuquerque, NM).

### Migration assay

Human fetal lung fibroblasts were plated in six well plates and incubated to reach confluence before being transfected with different siRNA in the presence or absence of rhFGF18 treatment. A straight line was drawn across the plate with a pipette tip. Images of the scratched cells were taken after 0- and 24-h using microscope (Leica DMi1, Leica Biosystems, Visa, CA). The migration ability by the cells was analyzed using ImageJ (Schindelin et al., 2012). Briefly, the closure area was calculated using the following formula: closure (%) = [(T_0_ - T_24_)/T_0_] x 100, where T_0_ represents the initial wound area and T_24_ represents the remaining area at 24 h.

### Western blot (WB) analyses

Following the different treatments, cells were lysed on ice in RIPA (Radioimmunoprecipitation assay) buffer supplemented with halt protease (Thermofisher) and phosphatase cocktail inhibitors (P5726 and P0044, Sigma). Proteins were quantified using DC protein assay kit (Biorad, Hercule, CA, USA) according to the manufacturer’s instructions. 20 μg of protein were loaded on an 8% Bis-Tris Plus precast polyacrylamide gel and run in 1X Bolt MES SDS running buffer (Invitrogen) using the Bolt System (Invitrogen Waltham, MA, USA). Transfers were performed using the iBlot 2 System (Invitrogen) and nitrocellulose gel transfer stacks. After the transfer, membranes were blocked in a 50:50 Odyssey Blocking (LI-COR, Lincoln, NE, USA): TBST (TBS and 0.1% Tween) solution at RT for at least 1 h and incubated with primary antibodies for 24 h at 4°C with continuous agitation (Table 4). The following day, membranes were washed with TBST and incubated with fluorescent secondary antibodies diluted in blocking buffer for 1 h at RT. Final detection was obtained by enhanced fluorescence with Chemidoc MP imaging system (Biorad, Hercules, CA, USA). Densitometry was analyzed using ImageLab software (Version 6.1, Biorad).

### Statistical analyses

Statistical analyses were performed using GraphPad Prism (GraphPad Software Inc. La Jolla, CA, USA). Normality was assessed for each group using the Shapiro-Wilk test. If a group passed assumptions for parametric testing, a one-way ANOVA test was employed to compare each experiment to the control. The resulting *p*-values were corrected using the Dunnett test. If data did not pass the Shapiro-Wilk test, a Kruskal–Wallis test was conducted. The resulting *p*-values were adjusted using Dunn’s test. To compare two non-control groups (e.g., SiFGFR2 to SIFGFR2-FGF18), a *t*-test was performed if the data passed parametric assumptions. Furthermore, a Mann-Whitney test was used to compare two non-control samples when parametric testing was not applicable. The results were considered significant if *p* ≤ 0.05.

## Results

### Stage-specific modulation of mesenchymal proliferation in human lung development by FGF18 and its receptors

**FIGURE 1:**
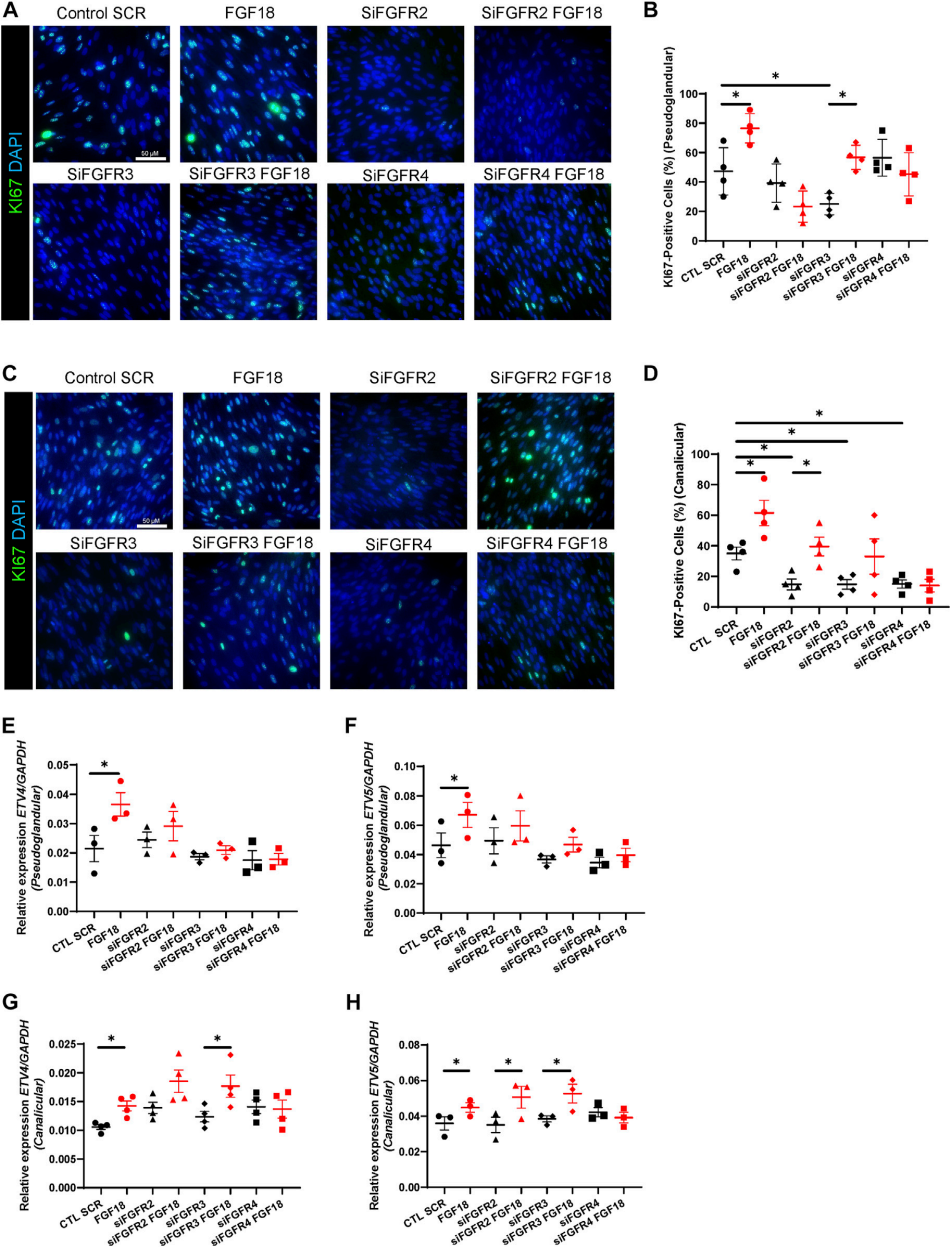
Activation of FGFR2/FGFR4 in pseudoglandular stage and FGFR3/FGFR4 in canalicular stage mediate FGF18-induced proliferation during human lung development. IF staining of KI67 (green) of pseudoglandular **(A)** or canalicular stage **(C)** human fetal lung fibroblasts (HFLF) treated or not with rhFGF18 alone, or 10 nM siRNA FGFR2 (siFGFR2), siRNA FGFR3 (siFGFR3) and siRNA FGFR4 (siFGFR4) supplemented or not with rhFGF18. Scramble siRNA (CTL SCR) was used as control. Quantification of total KI67-positive cells in pseudoglandular **(B)** and canalicular stage **(D)** HFLF. Results are shown as dot plot with mean ±SEM, *n* = 4 for each group. RT-qPCR for *ETV4* **(E)** and *ETV5* **(F)** in HFLF from pseudoglandular stage. RT-qPCR for *ETV4* **(G)** and *ETV5* **(H)** in HFLF from canalicular stage. Results are shown as dot plot with mean ±SEM, *p < 0.05, *n* = 3 for each group.

During human lung development, proliferation of mesenchymal cells is critical for branching morphogenesis and alveologenesis (Herriges and Morrisey, 2014) as defective proliferation can lead to congenital lung malformations and respiratory distress in newborns (Li et al., 2018). We first isolated and cultured human fetal lung fibroblasts (HFLF) from pseudoglandular (12–13 weeks) and canalicular (19–21 weeks) gestation lungs to evaluate the role of the FGF18/FGFR binding on cell proliferation. We previously demonstrated that FGF18 increases cell proliferation mainly in the epithelium of human explant cultures (Danopoulos et al., 2023). To determine whether FGF18 plays a role in cell proliferation in isolated lung mesenchymal cells at different gestational times, we treated HFLF with recombinant human protein FGF18 (rhFGF18). IF staining for KI67 demonstrated that FGF18 increased proliferation in HFLF independently of the age (pseudoglandular: 76.5% ± 4.9% vs. 47.25% ± 8%, *p* = 0.02, *n* = 4, Figure 1B; canalicular: 61% ± 8.2% vs. 35% ± 4.1%, *p* = 0.02, *n* = 4; Figure 1D). To identify the receptor through which FGF18 regulates HFLF proliferation, we first sought to investigate the expression of the different FGFRs in HFLF. We performed RT-qPCR to compare the baseline expression levels of the different FGFRs in both stages HFLF (Supplementary Figures S1A–C). The results showed no significant difference in the expression of *FGFR2, 3,* and *4* between pseudoglandular and canalicular stage HFLF. We next performed an siRNA assay for *FGFR2, 3,* and *4* to determine the ligand-receptor complex associated with proliferation. We confirmed successful downregulation of *FGFR2* (siFGFR2, *p* = 0.012 in pseudoglandular and *p* = 0.014 in canalicular stage; *n* = 4, Supplementary Figure S1A), *FGFR3* (siFGFR3, *p* = 0.003 in pseudoglandular and *p* = 0.018 in canalicular stage; *n* = 4, Supplementary Figure S1B) and *FGFR4* (siFGFR4, *p* = 0.049 in pseudoglandular and *p* = 0.025 in canalicular stage; *n* = 4, Supplementary Figure S1C) by RT-qPCR as compared to the scrambled control (CTL SCR). The addition of rhFGF18 (100 ng/mL) did not alter the silencing effect (Supplementary Figures S1A–C). The pseudoglandular stage HFLF were treated with the different receptor siRNA (100 nM) in presence or absence of rhFGF18 for 24 h (Figures 1A, B) and stained with KI67 to assess proliferation. siRNA treatment for *FGFR2* and *FGFR4* did not affect proliferation, however, siRNA for *FGFR3* significantly decreased the proliferation as compared to control (25% ± 3.6% vs. 47.25% ± 8%, *p* = 0.02; *n* = 4, Figure 1B). The addition of rhFGF18 following siRNA *FGFR3* treatment rescued the decrease in cell proliferation to baseline control (56.7% ± 4.1% vs. 25% ± 3.6%, *p* = 0.01; *n* = 4, Figure 1B). However, the combination of siRNA for *FGFR2* or *FGFR4* treatments with rhFGF18 did not produce any significant rescue in proliferation compared to baseline control. The results suggested a role of rhFGF18 in pseudoglandular stage HFLF through FGFR2 and FGFR4. Canalicular stage HFLF were treated with the same conditions (Figures 1C, D). siRNA treatment for *FGFR2, 3* or *4* significantly reduced HFLF proliferation (*p* = 0.03, *p* = 0.04 and *p* = 0.04 respectively; *n* = 4; Figure 1D). The addition of rhFGF18 only partially rescued the proliferation rate to baseline control when combined with siRNA targeting *FGFR2* (33% ± 9% vs. 14.7% ± 3.5%, *p* = 0.04; *n* = 4; Figure 1D). These results suggested that rhFGF18 may have a role in promoting HFLF proliferation during the canalicular stage through *FGFR3* and *FGFR4*. RT-qPCR analysis were performed to evaluate the expression levels of *ETV4* and *ETV5* transcription factors (Figures 1E–H), downstream targets of the FGF pathway that are frequently associated with cell proliferation (Herriges et al., 2015). Treatment with rhFGF18 led to a significant increase in the expression levels of *ETV4* and *ETV5* in both stage HFLF as compared to CTL SCR (respectively for pseudoglandular *p* = 0.04 and *p* = 0.02; and for canalicular *p* = 0.04 and *p* = 0.02; *n* = 3; Figures 1E–H). The expression of these transcription factors was not affected by the siRNA treatment; however, in pseudoglandular stage we noted that none of the siRNA rescue the expression of *ETV4* and *ETV5* after rhFGF18 treatment. Conversely, canalicular stage HFLF treated with siRNA for *FGFR2* or *FGFR3* and supplemented with rhFGF18, showed restored expression of *ETV4* and *ETV5* (respectively 0.03 ± 0.004 vs. 0.02 ± 0.004, *p* = 0.04 and 0.06 ± 0.008 vs. 0.04 ± 0.008, *p* = 0.02; *n* = 3; Figures 1G, H). No change was observed for *FGFR4* siRNA. Finally, we investigated the protein levels of phospho-ERK through Western blot analysis (p-ERK, Supplementary Figure S1D–F). ERK signaling acts as a downstream target of the FGF pathway, promoting proliferation through the activation of ETVs transcription factors (Xiao et al., 2012; Joannes et al., 2016; Garg et al., 2020). Our findings demonstrate a significant increase in relative p-ERK following FGF18 treatment in both pseudoglandular and canalicular stages as compared to control (respectively 0.189 ± 0.014 vs. 0.105 ± 0.034, *p* = 0.048; Supplementary Figure S1E and 1.26 ± 0.16 vs. 0.92 ± 1.88, *p* = 0.005; Supplementary Figure S1F; *n* = 3). The addition of rhFGF18 after FGFR2 siRNA failed to induce ERK phosphorylation in pseudoglandular stage HFLF (Supplementary Figure S1E). A similar pattern was observed in the canalicular stage HFLF for FGFR4 (Supplementary Figure S1F). These results suggest that rhFGF18 enhances proliferation in both stages HFLF by upregulating *ETV*s transcription factor and ERK phosphorylation, through distinct FGFRs.

### FGF18 enhances HFLF migration from all stages

**FIGURE 2:**
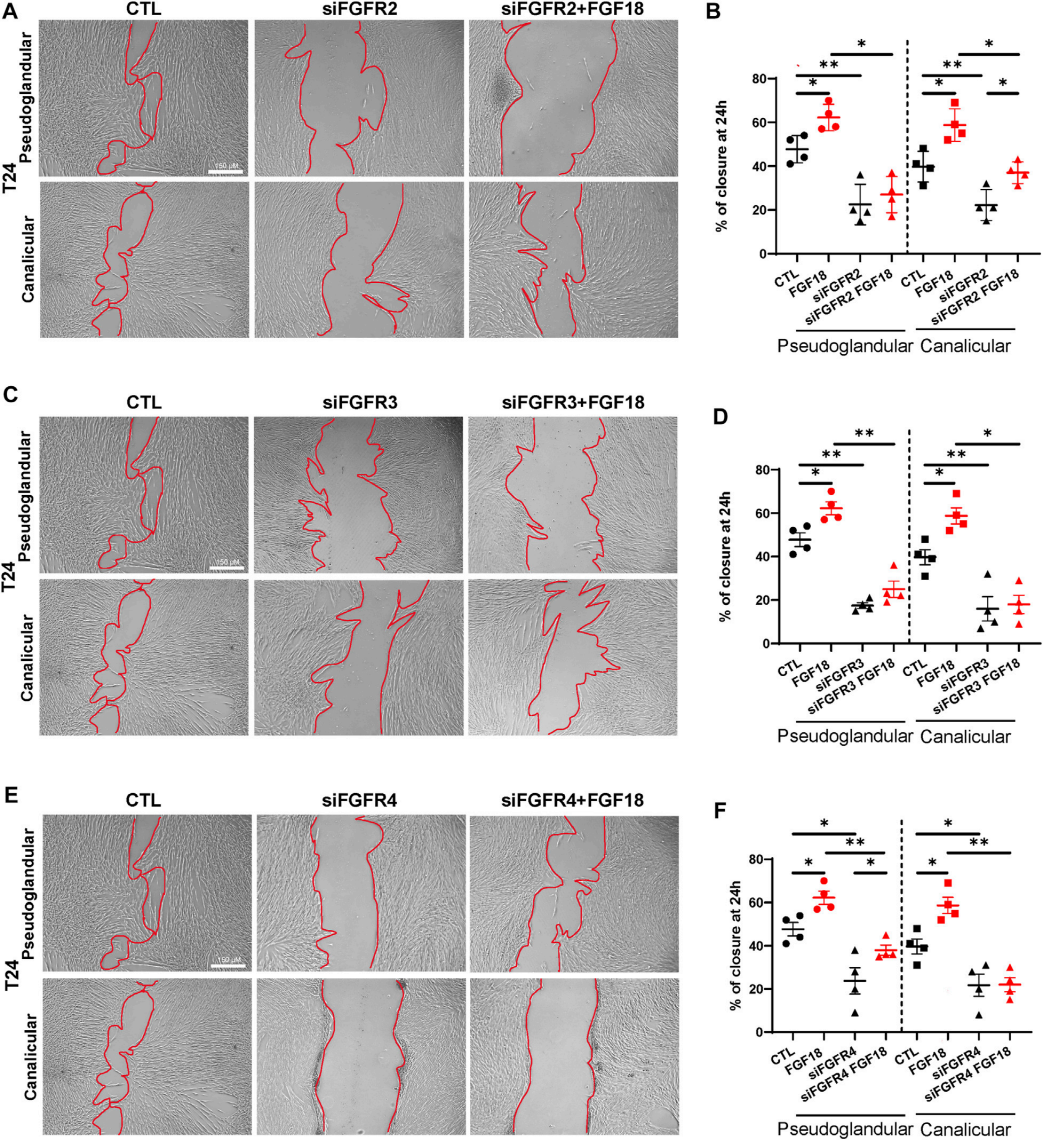
FGF18 enhances mesenchymal cell migration within lung development. Representative images of a scratch assay taken 24 h after 10 nM siRNA treatment for FGFR2 (siFGFR2), FGFR3 (siFGFR3) or FGFR4 (siFGFR4) supplemented or not with rhFGF18 **(A, C, E)**. The percentage of wound closure was quantified at 24 h **(B, D, F)**. Results are shown as dot plots with mean ±SEM, *p < 0.05, **p < 0.01, *n* = 4 for each group.

During human fetal lung development, mesenchymal cells migrate to provide structural support to the developing lung (Lee et al., 2017; Nasri et al., 2021). The migration of these cells is regulated by several factors, including FGF signaling (Joannes et al., 2016; El Agha et al., 2017). To identify the FGF18/FGFR complex(es) associated with this biological process, we performed scratch assays (Figure 2; Supplementary Figures S1G–I). We demonstrated that rhFGF18 significantly enhanced cell migration in both pseudoglandular and canalicular stage HFLF (respectively 55.2% ± 3.3% vs. 38.7% ± 6%; *p =* 0.01; and 29.1% ± 3.7% vs. 14.7% ± 2.3%; *p =* 0.04; *n* = 3; Supplementary Figures S1G,H). We noted that pseudoglandular stage HFLF presented a higher percentage of scratch closure at 24 h as compared to canalicular stage (*p* = 0.02). In addition, treatment with siRNA resulted in a significant reduction in migratory capacity of cells from both stages HFLF, as shown in Figures 2A, C, E. The percentage of wound closure was decreased by approximately 50% following siRNA treatment for all receptors (Figures 2B, D, F). In the pseudoglandular stage, the combination of FGFR2 siRNA and rhFGF18 did not restore HFLF migration (*p* = 0.51; *n* = 4; Figure 2B), while in the canalicular stage, HFLF migration was partially rescued (37% ± 2.4% vs. 22.2% ± 3.5%; *p =* 0.03; *n* = 4; Figure 2B). Interestingly, cells treated with FGFR3 siRNA and rhFGF18 failed to rescue the migration in both stages HFLF (Figure 2C). Finally, FGFR4 siRNA combined with rhFGF18 partially rescued the migratory capacities of pseudoglandular stage HFLF (38% ± 2.3% vs. 23.7% ± 6.1%, *p =* 0.03; *n* = 4; Figure 2F), whereas canalicular stage HFLF were unresponsive. The data indicated that rhFGF18 treatment enhanced cell migration in both pseudoglandular and canalicular stage HFLF through different receptors.

### Stage dependent FGF18/FGFR combinations influence mesenchymal differentiation

**FIGURE 3:**
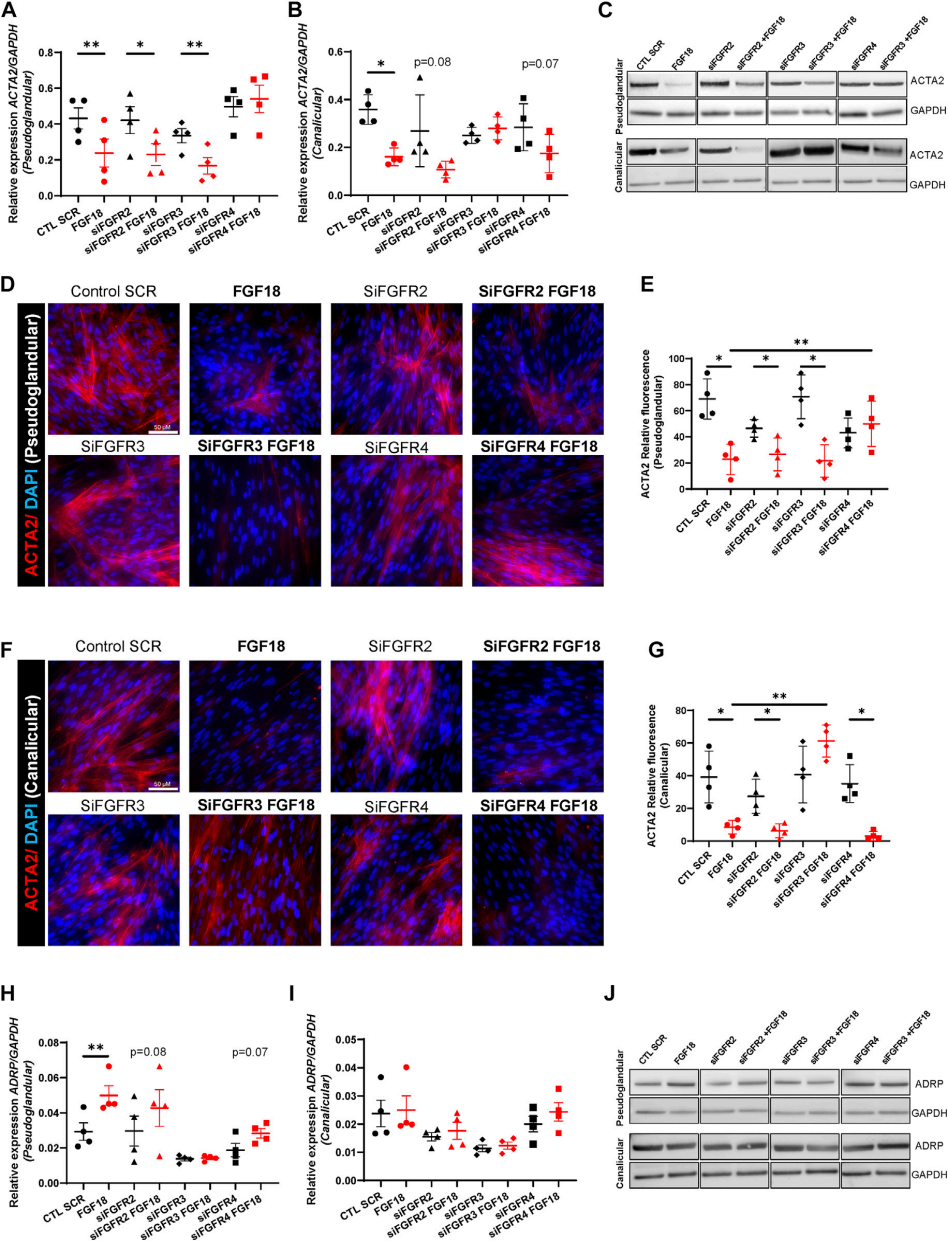
Specific FGF18/FGFR activity regulates mesenchymal cell differentiation. RT-qPCR for *ACTA2* in pseudoglandular **(A)** and canalicular stage **(B)** HFLF treated or not with 10 nM siRNA for *FGFR2* (siFGFR2), *FGFR3* (siFGFR3) or *FGFR4* (siFGFR4) complemented or not with rhFGF18 (in red). Representative western blots for ACTA2 and GAPDH in HFLF treated or not with siRNA and rhFGF18 **(C)**. IF staining of ACTA2 of pseudoglandular **(D)** or canalicular stage **(F)** HFLF treated or not with siRNA and rhFGF18. Relative fluorescent quantification was assessed (respectively **(E,G)**). RT-qPCR for *ADRP* in pseudoglandular **(H)** and canalicular stage **(I)** HFLF. Representative western blots for ADRP and GAPDH in HFLF **(J)**. Results are shown as dot plots with mean ±SEM, *p < 0.05, **p < 0.01, *n* = 4 for each group.

We recently demonstrated that FGF18 promotes human lung branching morphogenesis through regulation of mesenchymal progenitor cells (Danopoulos et al., 2023), but the underlying mechanisms remain unclear. Previous studies showed that during early embryonic mouse lung development, mesenchymal progenitor cells differentiate into myofibroblasts, while in later stages, they differentiate into lipofibroblasts (Al Alam et al., 2015; Riccetti et al., 2020; Liu et al., 2021). Therefore, we investigated the effect of the FGF18/FGFR pathway on mesenchymal cell fate. For this purpose, we assessed myofibroblast (ACTA2, COL1A1) and lipofibroblast (ADRP, PPARγ) markers in pseudoglandular and canalicular HFLF treated with rhFGF18 (Figure 3). RT-qPCR analysis revealed that treatment with rhFGF18 resulted in decreased *ACTA2* expression in both pseudoglandular and canalicular stage HFLF (0.23 ± 0.07 vs. 0.4 ± 0.05, *p =* 0.006 and 0.16 ± 0.01 vs. 0.35 ± 0.03, *p* = 0.01 respectively, *n* = 4; Figures 3A, B). Furthermore, treatments with *FGFR2, FGFR3* or *FGFR4* siRNA did not alter *ACTA2* expression from baseline expression (Figures 3A, B). Whereas the use of FGFR2 or FGFR3 siRNA combined with rhFGF18 significantly decreased *ACTA2* gene expression in pseudoglandular stage HFLF (0.23 ± 0.06 vs. 0.42 ± 0.07, *p* = 0.02 and 0.16 ± 0.04 vs. 0.33 ± 0.03, *p* = 0.009 respectively; n = 4; Figure 3A), the combination of rhFGF18 with FGFR4 siRNA had no effect on *ACTA2* expression. A decrease in ACTA2 protein levels was also observed by WB (Figure 3C, top panel, and Supplementary Figures S2A,B) (Figures 3D, E). Similar to what was observed in pseudoglandular stage, canalicular stage HFLF displayed a decreasing trend of *ACTA2* expression when treated with FGFR2 siRNA and rhFGF18 (*p* = 0.08; Figure 2B), whereas FGFR3 siRNA combined with rhFGF18 showed no change. These results were also observed at the protein level, as demonstrated by WB and IF staining (Figure 3C, bottom panel; Figures 3F, G). In contrast to what was observed in pseudoglandular stage, HFLF treated with FGFR4 siRNA in conjunction with rhFGF18 showed a significant decrease in *ACTA2* expression (0.15 ± 0.03 vs. 0.28 ± 0.04; *n* = 4, *p* = 0.02; Figure 3B), which was confirmed by WB (Figure 3C, bottom panel) and IF staining quantification (Figures 3F, G). The regulation of *COL1A1*, another myofibroblast marker, was also investigated (Supplementary Figures S2C,D). *COL1A1* expression was significantly reduced after rhFGF18 treatment in both stages (3.42 ± 0.4 vs. 6.17 ± 0.6, *p* = 0.03, *n* = 4 in pseudoglandular stage and 2.06 ± 0.3 vs. 5.45 ± 0.4 in canalicular stage HFLF, *p* = 0.003, *n* = 3 Supplementary Figures S2C,D). However, the addition of rhFGF18 to the siRNA treatment did not lead to a similar decrease in both stages.

We then assessed the expression of *ADRP* (lipofibroblast marker) in the same conditions (Figures 3H–J). rhFGF18 significantly increased *ADRP* expression solely in pseudoglandular stage HFLF (0.04 ± 0.05 vs. 0.02 ± 0.004; *n* = 4; *p =* 0.001; Figures 3H, I). Although no significant differences were observed following treatment with siRNA and rhFGF18, we did observe a trend of upregulation in the pseudoglandular stage in the presence of FGFR2 and FGFR4 siRNA (Figure 3H). These results were confirmed by WB (Figure 3J and S2E and F). Moreover, *PPARγ* (lipofibroblast marker) expression was significantly upregulated in both stages HFLF by rhFGF18 (respectively 0.005 ± 0.0004 vs. 0.002 ± 0.0004; *p* = 0.01, and 0.001 ± 0.0001 vs. 0.008 ± 0.00005, *p* = 0.02; *n* = 3; Supplementary Figures S2G,H). *PPARγ* gene expression demonstrated an increasing trend in both pseudoglandular and canalicular stage cells treated with FGFR2 siRNA supplemented by rhFGF18, whereas a significant upregulation was observed for FGFR3 siRNA treated with rhFGF18 in the pseudoglandular stage only (0.003 ± 0.0005 vs. 0.002 ± 0.004; *p* = 0.008; *n* = 3; Supplementary Figure S2H). Combining FGFR4 siRNA with rhFGF18 did not produce the same results as rhFGF18 alone (Supplementary Figures S2G,H). Taken together, our data suggest that FGF18/FGFR(s) plays a role in promoting the differentiation of mesenchymal progenitors by reducing the expression of myofibroblast markers and increasing lipofibroblast markers. However, the effects seem to vary depending on the stage and the specific ligand/receptor interaction.

## Discussion

**FIGURE 4:**
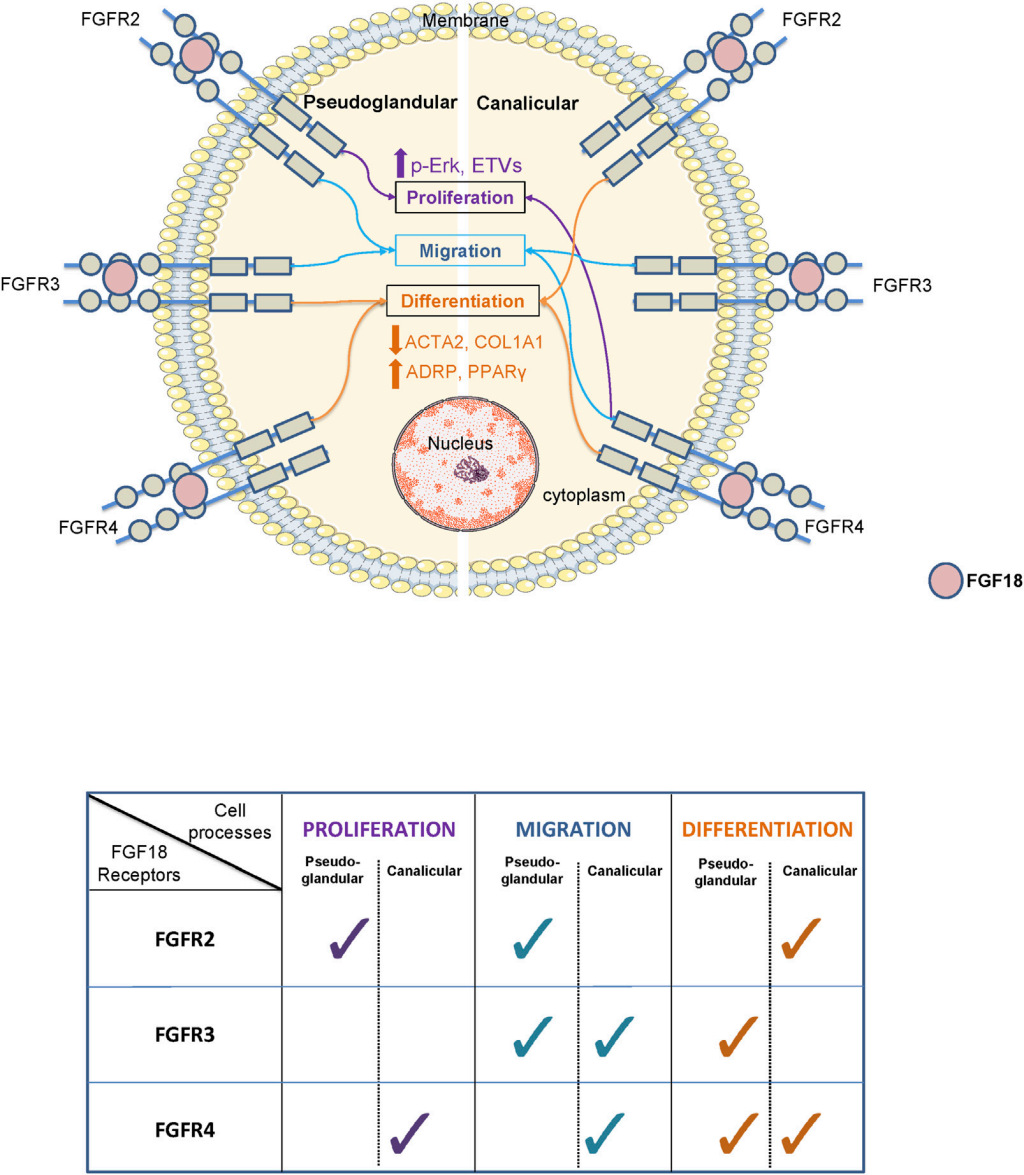
Schematic and table summarizing the role of FGF18 through its different receptors during the pseudoglandular and canalicular stage of human lung development.

For several decades, researchers have been attempting to comprehend the function of various signaling pathways in the process of development. Among them, the FGF pathway plays a critical role in the organogenesis of several organs including the lung. However, the underlying mechanisms remain poorly understood (Danopoulos et al., 2019a; Yuan et al., 2020; Yang et al., 2021). Several studies in mice demonstrated the role of FGF1, 7, 9 or 10 in different processes such as lung growth, cell-cell interaction, or differentiation (Cardoso et al., 1997; Park et al., 1998; Colvin et al., 2001). However, we recently established significant differences in FGF signaling between humans and mice (Danopoulos et al., 2019b). Our previous studies show that FGF18 is the factor responsible for promoting lung branching and regulating mesenchymal progenitor cells in humans, which differs from mice where FGF10 is the main morphogen regulating branching (Yuan et al., 2018; Danopoulos et al., 2019a; Danopoulos et al., 2023). The aim of this study was to investigate the mechanism by which FGF18 and its receptors (FGFRs) influence mesenchymal cells during human lung development, summarized in the schematic and table in Figure 4. We isolated HFLF from pseudoglandular (12–13 weeks) to canalicular (19–21 weeks) stage of lung development and investigated the impact of FGF18 on proliferation, migration, and differentiation. Additionally, we aimed to identify the FGF18/FGFR complex involved in proliferation, migration, and differentiation at each developmental stage.

We previously showed that human fetal lung explants treated with FGF18 presented an increase in epithelial proliferation, while no change was noted for the mesenchymal cells in pseudoglandular stage (Danopoulos et al., 2023). In this study, it was observed that isolated mesenchymal cells responded differently to FGF18 treatment, which implies that epithelial cells may play a crucial role in regulating the behavior of the mesenchymal compartment. Moreover, previous research on mice revealed that FGF18 deficiency led to reduced proliferation during the saccular stage (Usui et al., 2004), while a separate study showed that FGF18 suppressed isolated fibroblast proliferation during alveologenesis (McGowan and McCoy, 2015), emphasizing the significance of examining inter-species signaling variations and the value of investigating human models. Furthermore, although HFLF cells treated with rhFGF18 showed a significant increase in KI67 staining during both developmental stages, their proliferation was differentially impacted when the receptors were silenced by siRNA. Depending on the stage, the addition of rhFGF18 rescued the proliferation only after siRNA FGFR3 treatment in the pseudoglandular stage, and siRNA FGFR2 in the canalicular stage. These data suggested that FGF18 promotes mesenchymal cell proliferation through FGFR2 and FGFR4 during the pseudoglandular stage, while FGFR3 and FGFR4 appear to be involved in the canalicular one. This corroborates previous studies showing that FGF18 binds FGFR3 and FGFR4 to influence mesenchymal cells during late-stage of development (Srisuma et al., 2010; McGowan and McCoy, 2015), as well as in the adult human lungs (Joannes et al., 2016). Our knowledge of the role played by the FGF18/FGFR2 complex is limited, and our findings herein provide valuable insights that could enhance our understanding of its function in human lung development. Prior research has established the importance of the FGFR2/FGF10 complex in lung development in mice (Bellusci et al., 1997a; Herriges et al., 2015). Our recent research has revealed that FGF18 has a comparable function in human lung development as FGF10 in mouse lung development. Furthermore, we demonstrated an upregulation of the FGF downstream targets ETV4 and ETV5 following treatment with rhFGF18 during human fetal lung development, as well as an increase of p-ERK levels. In the pseudoglandular stage, FGF18 may regulate ETV4/5 expression via all three types of receptors, while in the canalicular stage, distinct receptors may modulate ETVs expression. Previous research has identified ETV4/5 as important transcription factors in mouse lung development through the modulation of FGF10 signaling (Herriges et al., 2015; Zhang et al., 2016). ETVs transcription factors are often associated with ERK signaling, which has been shown to be activated via the FGF pathway to induce proliferation and migration in the lung (Joannes et al., 2016). Overall, these results suggests that FGF18 may enhance mesenchymal proliferation in through pseudoglandular to canalicular stage of human lung development via distinct receptors.

By conducting scratch assays to investigate mesenchymal cell migration, we found that treating cells with rhFGF18 led to increased migration in both pseudoglandular and canalicular stage HFLF, and knockdown of FGFRs using siRNA significantly decreased this migratory capacity. Consistent with earlier findings, it appears that FGFR2 plays a role in mesenchymal cell migration during the pseudoglandular stage human lung development, while FGFR4 is involved in the canalicular stage migration. Interestingly, FGFR3 may be involved in migration throughout lung development. The role of FGF18 in lung mesenchymal cell migration is not yet fully determined, however, it was demonstrated that FGF18 mediates migration in several adult lung disease (Joannes et al., 2016; Chen et al., 2017; Yu et al., 2018). Previous studies have demonstrated that the FGF18/FGFR3 complex promotes migration of mouse myofibroblasts during alveologenesis (McGowan and McCoy, 2015; Ruiz-Camp and Morty, 2015). Herein, we propose that FGFR3 might play a potential role in the migration of mesenchymal cells at a much earlier stage of lung development. The importance of FGFR2/FGF10 in promoting cell proliferation and migration during mouse lung development has been extensively documented (Bellusci et al., 1997b; Abler et al., 2009). Given our proposal of FGF18 as an alternative to FGF10 in human lung branching morphogenesis, our findings seem to align with these established results. We and others have reported that FGFR4 is expressed by mesenchymal cells through lung development in both human and mouse models (Powell et al., 1998; Danopoulos et al., 2023). Additionally, prior studies have demonstrated that FGFR4 collaborates with FGFR3 to promote migration during postnatal lung development in mouse (Weinstein et al., 1998). However, our findings demonstrate a connection between the FGF18 ligand and the function of FGFR4 in this context.

Lastly, we investigated how FGF18/FGFR signaling affects mesenchymal differentiation and we observed that rhFGF18 treatment resulted in decreased expression of myofibroblast markers ACTA2 and COL1A1, while lipofibroblast markers ADRP and PPARγ were increased. rhFGF18 impact on mesenchymal differentiation was found to be stage-specific, but it appears that FGFR3 and FGFR4 predominantly regulate this process. Several studies reported an important role for FGF18 in mesenchymal differentiation, specifically in facilitating the conversion of myofibroblasts to lipofibroblasts (McGowan and McCoy, 2015; Wu et al., 2018; Hagan et al., 2020). Our data corroborate these findings as well as our previous reports demonstrating a role for FGF18 in promoting chondrogenic cell fate in human fetal lung explants (Danopoulos et al., 2023). Interestingly, the expression of ADRP was unchanged in canalicular stage, suggesting that other pathways may be necessary to drive such expression.

In this study we used a simplistic approach to determine how and through which receptors FGF18 affects specifically the mesenchymal cells from pseudoglandular to canalicular stage of human lung development. Epithelial-cell interactions were not considered in this study, although they play an important role in proper lung development. Further studies are needed to expand on the role of FGF18 in regulating myofibroblasts, lipofibroblasts and/or chondrogenic cell fate.

## Data Availability

The datasets presented in this study can be found in online repositories. The names of the repository/repositories and accession number(s) can be found in the article/Supplementary Material.

## Supplementary material

SUPPLEMENTARY FIGURE S1 RT-qPCR for *FGFR2* **(A)**, *FGFR3* **(B)** and *FGFR4* **(C)** in the pseudoglandular (black) and the canalicular stage (red) HFLF after respective siRNA treatment complemented by rhFGF18 (*n* = 4 for each group). Results are shown as mean ±SEM, **p* < 0.05, ***p* < 0.01, *n* = 4 for each group. Representative western blots for p-ERK and GAPDH in HFLF treated or not with siRNA and rhFGF18 **(D)**. Dot plots (mean ±SEM) of Western blot densitometry ratios for p-ERK normalized to GAPDH in pseudoglandular **(E)** and canalicular stage **(F)** HFLF treated or not with siRNA and rhFGF18. Results are shown as mean ±SEM, **p* < 0.05, ***p* < 0.01, *n* = 3. Representative scratch-assay images at time 0 and after 24 h of pseudoglandular and canalicular stage HFLF treated or not with rhFGF18 **(G)**. Quantification of the wound closure at 24 h **(H)** in the pseudoglandular (black) and canalicular stage (red; *n* = 3 for each group). Results are shown as mean ±SEM, **p* < 0.05, ***p* < 0.01. Representative scratch-assay images at time 0 for siRNA treatments **(I)**.

SUPPLEMENTARY FIGURE S2 Dot plots (mean ±SEM) of Western blot densitometry ratios for ACTA2 in pseudoglandular **(A)** and canalicular stage **(B)** HFLF normalized to GAPDH treated or not with siRNA and rhFGF18. RT-qPCR for COL1A1 in pseudoglandular **(C)** and canalicular stage **(D)** HFLF treated or not with siRNA and rhFGF18. Dot plots (mean ±SEM) of Western blot densitometry ratios for ADRP in pseudoglandular **(E)** and canalicular stage **(F)** HFLF normalized to GAPDH. RT-qPCR for *PPARγ* in pseudoglandular **(G)** and canalicular stage **(H)** HFLF treated or not with siRNA and rhFGF18. Results are shown as mean ±SEM, **p* < 0.05, ***p* < 0.01, *n* = 3.
