# Supplementary figures and images for: Preferential FGF18/FGFR activity in pseudoglandular versus canalicular stage human lung fibroblasts
Source: Front Cell Dev Biol. 2023 Aug 28;11:1220002. doi: 10.3389/fcell.2023.1220002 (PMC10493313; doi:10.3389/fcell.2023.1220002)

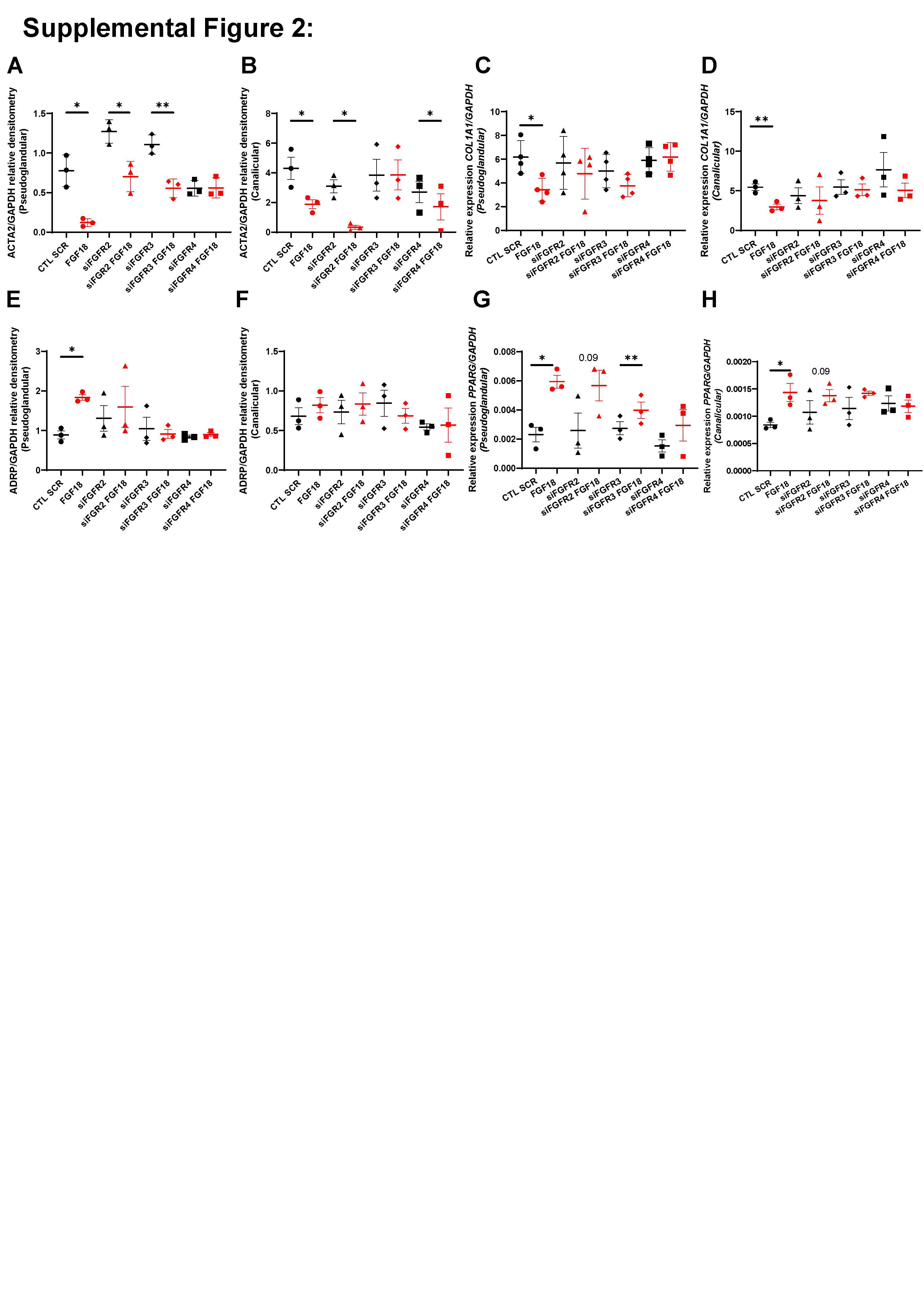

Supplement: Supplementary file 1 [file Image2.TIF]

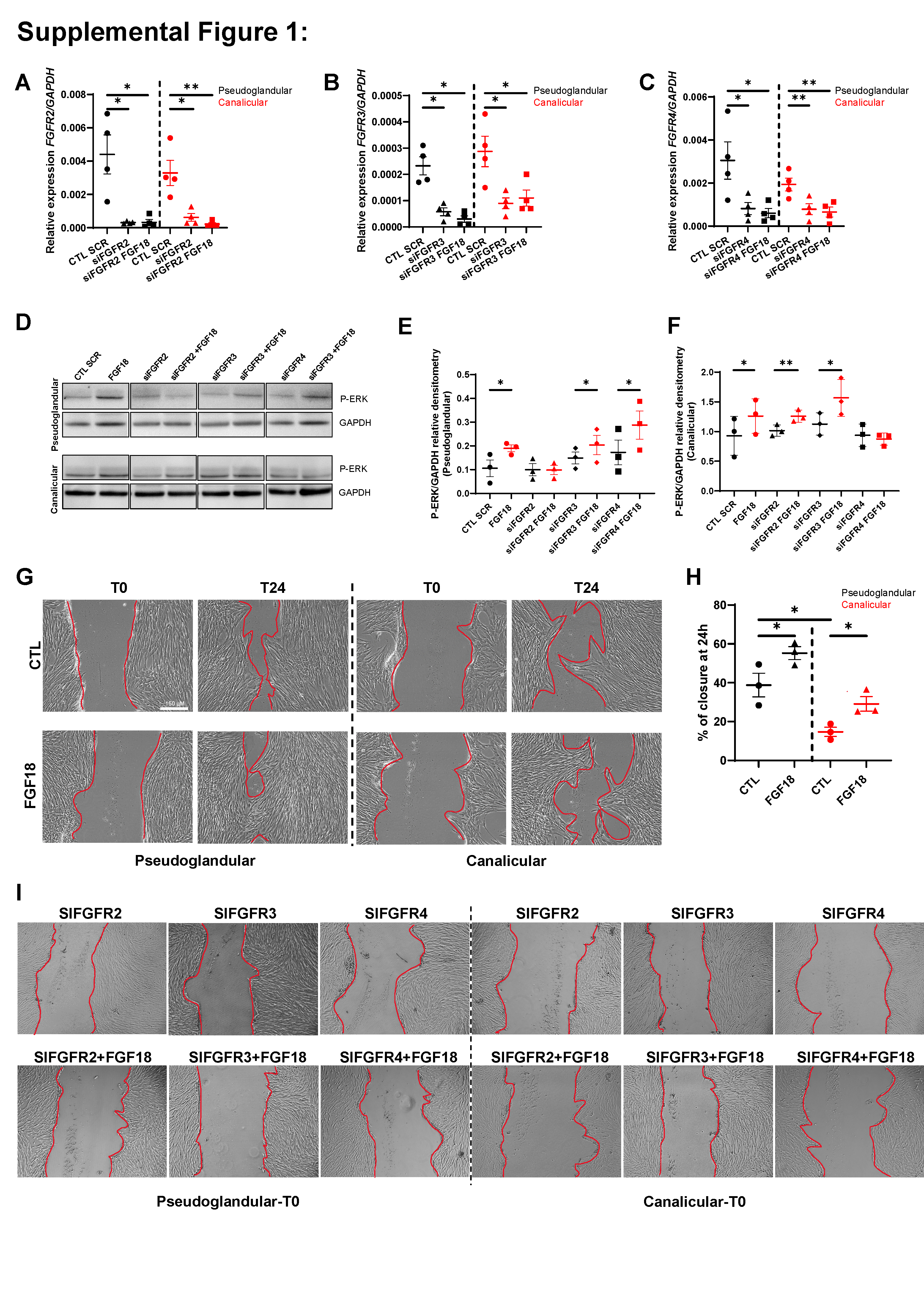

Supplement: Supplementary file 2 [file Image1.TIF]
